# The Clinical Profile of Unilateral and Bilateral Optic Disc Swelling in a Tertiary Center in Northern Malaysia

**DOI:** 10.7759/cureus.30572

**Published:** 2022-10-22

**Authors:** Ahmad-Marwan Abdul Aziz, Abdul-Salim Ismail, Azhany Yaakub

**Affiliations:** 1 Department of Ophthalmology and Visual Science, School of Medical Sciences, Universiti Sains Malaysia, Kubang Kerian, MYS; 2 Department of Ophthalmology, Penang Hospital, Georgetown, MYS

**Keywords:** unilateral optic neuritis, bilateral optic neuritis, papillitis, atypical optic neuritis, optic disc swelling, idiopathic optic neuritis

## Abstract

Background

Optic disc swelling (ODS) is a pathological condition with a variety of causes, including optic neuritis (ON), anterior ischemic optic neuropathy, and papilledema. Determining the causes of ODS is critical due to the possibilities of vision- or life-threatening diseases, such as space-occupying lesions. This study aimed to investigate the clinical profile of unilateral and bilateral ODS in Penang Hospital, Malaysia.

Methodology

This retrospective, descriptive study was conducted in Penang Hospital. Medical records of patients who were diagnosed with ODS from June 2018 until June 2020 in Penang Hospital Eye Emergency Clinic were reviewed. We excluded patients who defaulted on subsequent three months of follow up and those with pseudo-ODS.

Results

ODS was diagnosed in 43 patients who were all included in the study. Majority were females 55.8% (n = 24), with age ranging from 16 to 78 years. ON contributed most (41.9%, n = 18), followed by non-arteritic anterior ischemic optic neuropathy (NA-AION) (34.9%, n = 15), and papilledema (9.3%, n = 4). Other causes (14%, n = 6) included diabetic papillitis (n = 1), hypertensive retinopathy (n = 1), and central retinal vein occlusion (n = 4). Poor mean initial visual acuity was seen in patients with ON (1.07 ± 0.68) and NA-AION (1.33 ± 0.67). ON showed better final visual outcomes compared to NA-AION at the one-year follow-up.

Conclusions

ON and NA-AION were identified as the two most common causes of ODS in Penang Hospital for both unilateral and bilateral presentations. Most cases presented with poor initial visual acuity. After one year of follow-up, good visual recovery was seen in ON cases compared to other cases. These results were comparable with studies conducted in other Asian counties.

## Introduction

Optic disc swelling (ODS) is a pathological condition with a variety of causes. Papilledema is an exclusive term used to indicate passive edema of the optic disc associated with increased intracranial pressure [1]. Optic nerve swelling from etiology other than increased intracranial pressure is known as optic disc edema. ODS can be unilateral or bilateral. The clinical causes associated with unilateral ODS are optic neuritis (ON), non-arteritic anterior ischemic optic neuropathy (NA-AION), compressive optic neuropathy, retinal vein occlusion, and diabetic papillopathy. Bilateral ODS cases are often associated with papilledema, infiltrative optic neuropathy, toxic optic neuropathy, and malignant hypertension [2]. A recent study in Egypt by El Saed et al. reported that the most common etiology of unilateral ODS is NA-AION [3]. A Malaysian study by Ismail et al. reported that papillitis was the most common type of ON documented, where more than 50% were idiopathic, and multiple sclerosis was relatively rare [4]. Determining the causes is critical in view of possible vision- or life-threatening diseases. This study aimed to determine the etiology, clinical manifestations, and outcomes of ODS in Penang Hospital.

## Materials and methods

This study was approved by the Research and Ethics Committee of Penang Hospital and registered under the National Medical Research Register. This descriptive, retrospective study was conducted in the Department of Ophthalmology, Penang Hospital. We adhered to the tenets of the Declaration of Helsinki. We retrospectively reviewed the clinical records of all consecutive patients with unilateral or bilateral ODS diagnosed at Ophthalmology Clinic of Penang Hospital between June 2018 and June 2020. We excluded patients who defaulted on subsequent three months of follow-up and those with pseudo-ODS. All patients were subjected to detailed ophthalmic examination, including visual acuity (VA), red saturation, bright sensitivity, color vision, and detailed slit lamp examination. Fundus examinations with a 90D lens were performed and photographs were obtained to determine the existence of ODS by ophthalmologists. For mild ODS or cases that were vague in fundus findings, we additionally performed optical coherence tomography and evaluated the thickness of the retinal nerve fiber layer.

The diagnosis of each disease was determined by the following criteria. Papilledema was diagnosed when optic disc edema was present with signs of elevated intracranial pressure. We defined ON using the criteria of the Optic Neuritis Treatment Trial (ONTT) [5]. Patients with NA-AION were recruited according to the Ischemic Optic Neuropathy Decompression Trial (IONDT) criteria [6]. For diabetic papillopathy, we defined patients with the presence of diabetes and optic disc edema, the absence of substantial optic nerve dysfunction, and the absence of evidence of ocular inflammation or elevated intracranial pressure [7]. Other diseases were diagnosed by their characteristic clinical features. VA at presentation was determined as initial VA, while VA at the one-year follow-up was determined as the final VA.

The data collected were recorded and analyzed using the SPSS version 26.0 (IBM Corp., Armonk, NY USA).

## Results

Our study included 56 eyes from 43 consecutive patients who were diagnosed with ODS. There were 48.8% Chinese (n = 21), 41.9% Malays (n = 18), and 9.3% Indian (n = 4). There were 55.8% female (n = 24) and 44.2% male (n = 19) patients, with age ranging from 16 to 78 years. The majority of the cases were ON (41.9%, n = 18), followed by NA-AION (34.9%, n = 15), papilledema (9.3%, n = 4). Other causes (14%, n = 6) included one case of diabetic papillopathy, one case of hypertensive retinopathy, and four cases of central retinal vein occlusion (Table 1).

**Table 1:** Demographic and clinical findings of different types of optic disc swelling. NA-AION = non-arteritic anterior ischemic optic neuropathy; ON = optic neuritis; PE = papilledema; VA = visual acuity; logMAR = logarithm of the minimum angle of resolution

Characteristic	Total (n = 43)	ON (n = 18)	NA-AION (n = 15)	PE (n = 4)	Others (n = 6)
Total, n (%)	43 (100)	18 (41.9)	15 (34.9)	4 (9.3)	6 (14.0)
Age (mean ± SD, years)	48.47 ± 16.11	39.71 ± 13.28	59.20 ± 9.41	31.25 ± 11.87	61.50 ± 11.86
Gender, n (%)					
Male	19 (44.2)	5 (27.8)	9 (60.0)	2 (50)	3 (50)
Female	24 (55.8)	13 (72.2)	6 (40.0)	2 (50)	3 (50)
Race, n (%)					
Malay	18 (41.9)	10 (55.6)	3 (20)	2 (50)	3 (50)
Chinese	21 (48.8)	7 (38.9)	10 (66.7)	2 (50)	2 (33.3)
India	4 (9.3)	1 (5.6)	2 (13.3)	-	1 (16.7)
Laterality, n (%)					
Unilateral	30 (69.8)	17 (77.8)	10 (66.7)	2 (50)	4 (66.7)
Bilateral	13 (30.2)	4 (22.2)	5 (33.3)	2 (50.0)	2 (33.3)
Pain, n (%)	21 (48.8)	11 (61.1)	4 (26.7)	4 (100)	2 (33.3)
Initial VA (mean ± SD, logMAR)	1.19 ± 0.74	1.07 ± 0.68	1.33 ± 0.67	0.59 ± 0.88	1.65 ± 0.78
Final VA (mean ± SD, logMAR)	0.72 ± 0.76	0.43 ± 0.61	0.86 ± 0.55	0.26 ± 0.25	1.62 ± 1.04

A comparison of major causes of ODS is shown in Table 1. Younger mean age was observed in ON (39.71 ± 13.28) and papilledema (31.25 ± 11.87). NA-AION and other causes involved the older age group. Ocular pain or headache was commonly seen in 61.1% of ON cases (n = 11) and 100% of papilledema cases (n = 4). Poor mean initial VA was seen in patients with ON (1.07 ± 0.68), NA-AION (1.33 ± 0.67), and other causes (1.65 ± 0.78). While patients with papilledema presented with mild visual disturbance for initial VA (0.59 ± 0.88) (Table 1).

Looking into unilateral ODS cases, as seen in Table 2, the majority were from ON patients (n = 14, 46.7% from total unilateral ODS), followed by NA-AION (n = 10, 33.3%). Two cases of papilledema that presented with unilateral ODS were both cases of Foster-Kennedy syndrome and unilateral ODS with the contralateral atrophic optic disc. While in bilateral cases, majorities were from NA-AION patients (n = 5, 38.5% from a total of 13 bilateral ODS cases). Followed by ON (n = 4, 30.7%), papilledema (n=2, 15.4%), and other causes included one hypertensive retinopathy and one diabetic papillopathy cases (n = 2, 15.4%) (Table 2).

**Table 2:** Causes of unilateral and bilateral optic disc swelling. NA-AION = non-arteritic anterior ischemic optic neuropathy; ON = optic neuritis; PE = papilledema; HPT retinopathy = hypertensive retinopathy; CRVO = central retinal vein occlusion

Cause	Unilateral ODS (n = 30)	Bilateral ODS (n = 13)
ON, n (%)	14 (46.7)	4 (30.7)
NA-AION, n (%)	10 (33.3)	5 (38.5)
PE, n (%)	2 (6.7)	2 (15.4)
Others, n (%)	4 (13.3)	2 (15.4)
HPT retinopathy, (n, %)	-	(1, 7.7)
Diabetic papillopathy, (n, %)	-	(1, 7.7)
CRVO, (n, %)	4 (13.3)	-

We compared ON cases with NA-AION cases, as shown in Table 3. There were significant differences in mean age (p < 0.001), where ON cases involved the younger age group (39.71 ± 13.28) while NA-AION involved the older age group (59.20 ± 9.41). Although there were more female patients in the ON group and more male patients in the NA-AION group, they were not statistically significant (p = 0.065). Both ON and NA-AION had more unilateral ODS compared to bilateral ODS. Complaints of ocular pain were significantly more common in ON cases (p = 0.050). The duration of symptoms was greater in NA-AION cases compared to ON (20.47 ± 15.29 vs. 10.28 ± 13.68; p = 0.022) (Table 3). Both ON and NA-AION cases had poor initial VA. Significant improvement of final VA was found in ON cases compared to NA-AION (0.43 ± 0.61 vs. 0.86 ± 0.55; p = 0.045) (Table 3).

**Table 3:** Comparisons between optic neuritis and non-arteritic anterior ischemic optic neuropathy. NA-AION = non-arteritic anterior ischemic optic neuropathy; ON = optic neuritis; VA = visual acuity; logMAR = logarithm of the minimum angle of resolution + p < 0.05 is considered statistically significant based on the independent t-test. ++ p < 0.05 is considered statistically significant based on the Pearson chi-square test.

Characteristic	ON (n = 18)	NA-AION (n = 15)	P-value
Age (mean ± SD, years)	39.71 ± 13.28	59.20 ± 9.41	<0.001^+^
Gender, n (%)			0.065^++^
Male	5 (27.8)	9 (60.0%)	
Female	13 (72.2)	6 (40.0%)	
Laterality, n (%)			0.491^++^
Unilateral	14 (77.8%)	10 (66.7%)	
Bilateral	4 (22.2%)	5 (33.3%)	
Ocular pain, n (%)	11 (61.1%)	4 (19.0%)	0.050^++^
Symptom duration (mean ± SD, days)	10.28 ± 13.68	20.47 ± 15.29	0.050^+^
Risk factors for vasculopathy disease, n (%)	6 (33.3%)	11 (73.3%)	0.022^++^
Initial VA (logMAR)	1.07 ± 0.68	1.33 ± 0.67	0.288^+^
Final VA (logMAR)	0.43 ± 0.61	0.86 ± 0.55	0.045^+^

For optic neuritis cases, the majority were idiopathic (n = 11, 61.1%), followed by neuroretinitis (n = 4, 22.2%), multiple sclerosis (MS) (n = 2, 11.1%), and nasopharyngeal carcinoma (NPC) infiltration (n = 1, 5.6%) (Table 4), affecting unilaterally in most cases. Most patients with ON had good final visual outcomes, except for neuroretinitis cases (1.16 ± 1.09) (Table 4).

**Table 4:** Demographic and clinical data on optic neuritis according to the underlying causes. ON = optic neuritis; MS = multiple sclerosis; NPC = nasopharyngeal carcinoma; logMAR = logarithm of the minimum angle of resolution + p < 0.05 is considered statistically significant based on the independent t-test. ++ p < 0.05 is considered statistically significant based on the Pearson chi-square test.

Variables	Total	Idiopathic	Neuroretinitis	MS	NPC Infiltration	P-value
Number of patients, n (%)	18 (100)	11 (61.1)	4 (22.2)	2 (11.1)	1 (5.6)	
Gender, n (%)						
Female	13 (72.2)	9 (81.8)	2 (50.0)	2 (100)	-	0.182^++^
Male	5 (27.8)	2 (18.2)	2 (50.0)	-	1 (100)	
Age of onset (mean ± SD, years)	39.71 ± 13.28	36.96 ± 13.79	45.5 ± 15.24	35.0 ± 1.41	50.0 ± 0	<0.001^+^
Ethnic, n (%)						
Malay	10 (55.6)	6 (54.5)	2 (50.0)	2(100)	-	0.702^++^
Chinese	7 (38.9)	4 (36.4)	2 (50.0)	-	1 (100)	
Indian	1 (5.5)	1 (9.1)	-	-	-	
Laterality, n (%)						
Unilateral	14 (77.8)	10 (90.9)	1 (25.0)	2 (100)	1(100)	0.038^++^
Bilateral	4 (22.2)	1 (9.1)	3 (70.0)	-	-	
Ocular pain, n (%)	11 (61.1)	7 (63.6)	2(50)	2(100)	-	0.379^++^
Initial VA (logMAR)	1.07 ± 0.68	1.15 ± 0.61	1.23 ± 1.02	0.61 ± 0.55	0.50 ± 0	0.008^+^
Final VA (logMAR)	0.43 ± 0.61	0.23 ± 0.02	1.16 ± 1.09	0.22 ± 0	0.2 ± 0	<0.001^+^

## Discussion

The demographic data showed that the majority of the patients were of Chinese ethnicity. This reflects the population distribution in Penang. According to the Department of Statistics Malaysia 2020, Chinese ethnic composition was the highest in Penang (44.9%) [8]. This study analyzed the etiology of ODS. Our study showed ON and NA-AION as the two major causes of ODS for both unilateral and bilateral presentation. This result is consistent with the studies reported by Jung et al. [9] and Hata et al. [10]. The difference is that the previous study showed NA-AION as the most common cause and ON as the second most cause of ODS.

As the two major causes of ODS in this study, it is important to differentiate between ON and NA-AION because improvement in visual function is different despite similar initial VA impairments. In this study, we only encounter NA-AION cases and did not encounter cases of arteritic AION. On comparing clinical characteristics between ON and NA-AION, we found that there was a significant difference in age group, associated ocular pain, duration of symptoms, risk factors of vasculopathy disease, and the final VA outcome.

The younger age group seen in ON cases and the female predominance were comparable to previous studies by Ismail et al. [4], the ONTT [5], and other Asian studies on idiopathic ON [9-13]. The older age group was seen in NA-AION cases. Significant numbers of patients with NA-AION had vasculopathy diseases such as diabetes mellitus, hypertension, and hyperlipidemia. These conditions may be associated with the higher age group seen in NA-AION cases.

A significantly shorter duration of symptoms was seen in ON cases compared to NA-AION. Similar findings were also reported by Jung et al [9]. In our study, although both cases described the symptoms as acute onset, NA-AION arrived late to seek medical advice due to requiring spouses or relatives to bring them to the hospital. While younger ON patients tended to seek medical advice faster.

Our study revealed that ON was associated with better final visual outcomes compared to NA-AION. The poor visual prognosis associated with NA-AION might be due to irreversible changes in the optic disc that resulted from insufficient optic disc blood supply [9].

For bilateral ODS, the majority of the cases were from NA-AION which is in contrast to other studies such as Urfalioglu et al. [14], Iijima et al. [15], and Abbas et al. [16], who reported papilledema as the most common cause of bilateral ODS. In our study, only four cases of papilledema were diagnosed in our clinic. This might be due to most of the papilledema cases diagnosed being either referred from neuromedical or neurosurgery department as an inpatient, which was not recorded in our clinic records.

Limitations of this retrospective study based on clinic follow-up include a small sample size as it was conducted in one institution and patients who were referred through the ward may not be recorded. In the future, we will create a data bank with multicenter involvement for a larger number of patients.

## Conclusions

ON and NA-AION were identified as the two most common causes of ODS in Penang Hospital for both unilateral and bilateral presentation. Most patients with ODS presented with poor initial VA except in papilledema cases which presented with mild VA disturbance. After one year of follow-up, good visual recovery was seen in ON cases compared to other cases. Our study is comparable to previous studies and is going to be practical for approaching ODS patients who present in our population at a local clinic.
